# Supplementary material for: Saccharomyces boulardii (CNCM I-745) improves intestinal damage in sepsis by remodeling intestinal flora structure
Source: Front Cell Infect Microbiol. 2025 Jul 31;15:1602792. doi: 10.3389/fcimb.2025.1602792 (PMC12350329; doi:10.3389/fcimb.2025.1602792)
Supplement: Supplementary file 1 [file DataSheet1.zip › summary/summary/8_Advanced_analyse/2_PICRUSt2_result/COG/Group.BW0_vs_AW0/PICRUSt2_COG_Group.BW0_vs_AW0_diff.pdf]

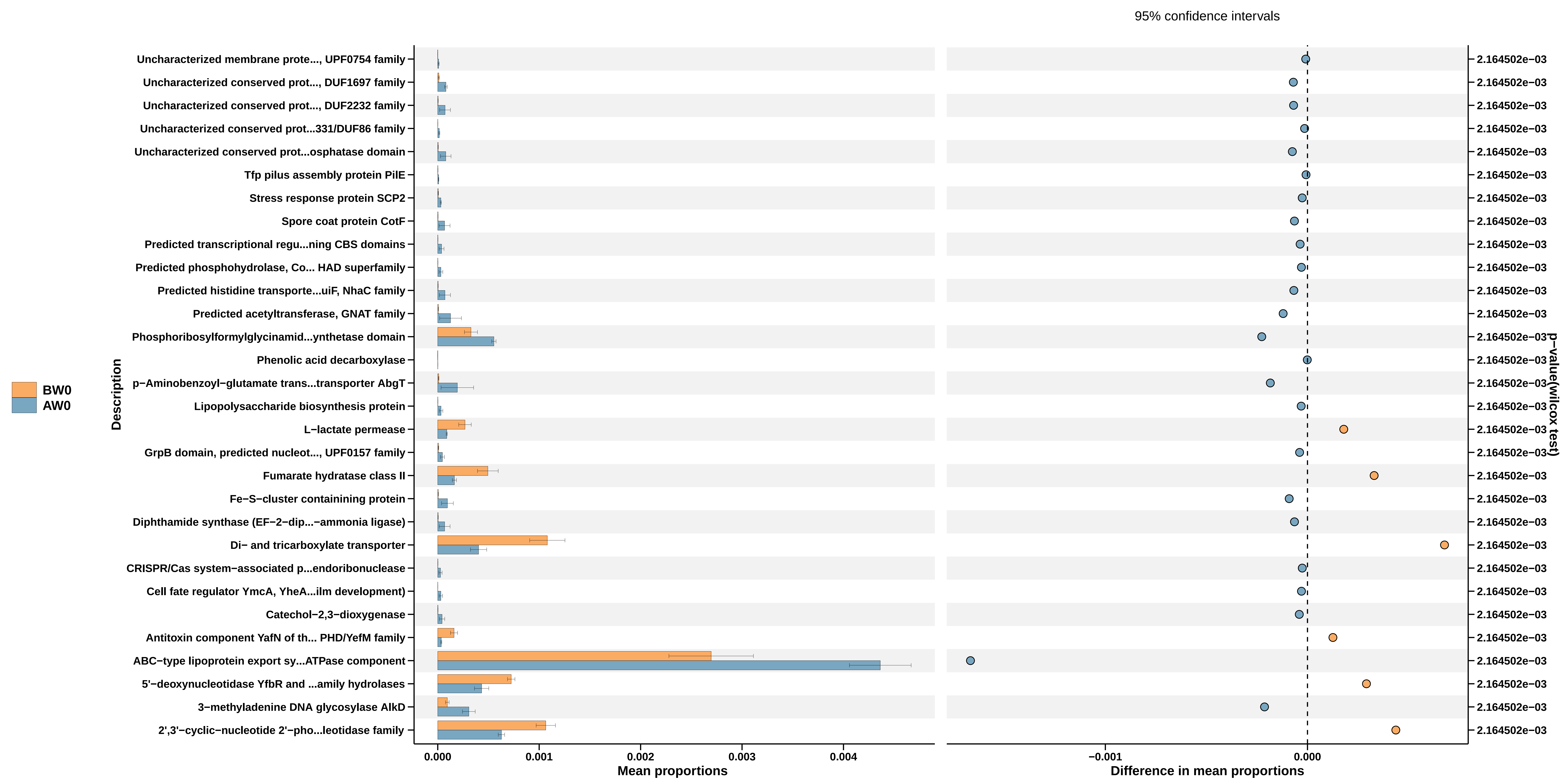
95% confidence intervals
BW0
AW0
Description
Uncharacterized membrane prote..., UPF0754 family
Uncharacterized conserved prot..., DUF1697 family
Uncharacterized conserved prot..., DUF2232 family
Uncharacterized conserved prot...331/DUF86 family
Uncharacterized conserved prot...osphatase domain
Tfp pilus assembly protein PilE
Stress response protein SCP2
Spore coat protein CotF
Predicted transcriptional regu...ning CBS domains
Predicted phosphohydrolase, Co... HAD superfamily
Predicted histidine transporte...uiF, NhaC family
Predicted acetyltransferase, GNAT family
Phosphoribosylformylglycinamid...ynthetase domain
Phenolic acid decarboxylase
p−Aminobenzoyl−glutamate trans...transporter AbgT
Lipopolysaccharide biosynthesis protein
L−lactate permease
GrpB domain, predicted nucleot..., UPF0157 family
Fumarate hydratase class II
Fe−S−cluster containining protein
Diphthamide synthase (EF−2−dip...−ammonia ligase)
Di− and tricarboxylate transporter
CRISPR/Cas system−associated p...endoribonuclease
Cell fate regulator YmcA, YheA...ilm development)
Catechol−2,3−dioxygenase
Antitoxin component YafN of th... PHD/YefM family
ABC−type lipoprotein export sy...ATPase component
5'−deoxynucleotidase YfbR and ...amily hydrolases
3−methyladenine DNA glycosylase AlkD
2',3'−cyclic−nucleotide 2'−pho...leotidase family
0.000
0.001
0.002
0.003
0.004
Mean proportions
−0.001
0.000
Difference in mean proportions
p−value(wilcox test)
2.164502e−03
